# Supplementary material for: Modeling glioblastoma heterogeneity as a dynamic network of cell states
Source: Mol Syst Biol. 2021 Sep 16;17(9):e10105. doi: 10.15252/msb.202010105 (PMC8444284; doi:10.15252/msb.202010105)
Supplement: Supplementary file 5 — Source Data for Figure 3 [file MSB-17-e10105-s001.zip › Figure3A_sourcedata/GSEA_3065/nosigdb_state6.GseaPreranked.1622718443993/RICHARDS_NOWAKOWSKI_SCIENCE_2017_RG_EARLY_UPREG.html]

Details for gene set RICHARDS\_NOWAKOWSKI\_SCIENCE\_2017\_RG\_EARLY\_UPREG[GSEA]

|  || Dataset | state6 |
| Phenotype | NoPhenotypeAvailable |
| Upregulated in class | na\_pos |
| GeneSet | RICHARDS\_NOWAKOWSKI\_SCIENCE\_2017\_RG\_EARLY\_UPREG |
| Enrichment Score (ES) | 0.35115448 |
| Normalized Enrichment Score (NES) | 1.797174 |
| Nominal p-value | 0.0 |
| FDR q-value | 0.0057727327 |
| FWER p-Value | 0.084 |
Table: GSEA Results Summary

  

Fig 1: Enrichment plot: RICHARDS\_NOWAKOWSKI\_SCIENCE\_2017\_RG\_EARLY\_UPREG      
 Profile of the Running ES Score & Positions of GeneSet Members on the Rank Ordered List

  

| PROBE | GENE SYMBOL | GENE\_TITLE | RANK IN GENE LIST | RANK METRIC SCORE | RUNNING ES | CORE ENRICHMENT || 1 | RPLP1 |  |  | 6 | 0.329 | 0.0074 | Yes |
| 2 | EEF1A1 |  |  | 9 | 0.325 | 0.0152 | Yes |
| 3 | RPLP0 |  |  | 10 | 0.312 | 0.0229 | Yes |
| 4 | RPS19 |  |  | 14 | 0.295 | 0.0299 | Yes |
| 5 | RPL37 |  |  | 19 | 0.287 | 0.0365 | Yes |
| 6 | RPL13 |  |  | 21 | 0.286 | 0.0435 | Yes |
| 7 | RPS28 |  |  | 24 | 0.283 | 0.0502 | Yes |
| 8 | RPLP2 |  |  | 31 | 0.271 | 0.0562 | Yes |
| 9 | RPL30 |  |  | 34 | 0.260 | 0.0624 | Yes |
| 10 | RPL18 |  |  | 36 | 0.256 | 0.0687 | Yes |
| 11 | RPL6 |  |  | 38 | 0.254 | 0.0748 | Yes |
| 12 | RPL18A |  |  | 39 | 0.252 | 0.0811 | Yes |
| 13 | RPL41 |  |  | 42 | 0.249 | 0.0870 | Yes |
| 14 | RPL29 |  |  | 47 | 0.247 | 0.0926 | Yes |
| 15 | TPT1 |  |  | 48 | 0.247 | 0.0987 | Yes |
| 16 | RPS8 |  |  | 51 | 0.245 | 0.1046 | Yes |
| 17 | FTH1 |  |  | 52 | 0.245 | 0.1106 | Yes |
| 18 | RPS3 |  |  | 59 | 0.238 | 0.1158 | Yes |
| 19 | RPL32 |  |  | 69 | 0.231 | 0.1204 | Yes |
| 20 | RPS14 |  |  | 72 | 0.229 | 0.1258 | Yes |
| 21 | RPL7A |  |  | 76 | 0.226 | 0.1311 | Yes |
| 22 | RPS21 |  |  | 77 | 0.226 | 0.1367 | Yes |
| 23 | RPL19 |  |  | 78 | 0.225 | 0.1422 | Yes |
| 24 | PEA15 |  |  | 79 | 0.225 | 0.1478 | Yes |
| 25 | RPS3A |  |  | 80 | 0.224 | 0.1533 | Yes |
| 26 | RPL39 |  |  | 82 | 0.223 | 0.1588 | Yes |
| 27 | RPS25 |  |  | 84 | 0.223 | 0.1641 | Yes |
| 28 | NACA |  |  | 86 | 0.221 | 0.1695 | Yes |
| 29 | RPL5 |  |  | 90 | 0.218 | 0.1745 | Yes |
| 30 | RPS27L |  |  | 91 | 0.218 | 0.1799 | Yes |
| 31 | SERF2 |  |  | 97 | 0.215 | 0.1847 | Yes |
| 32 | RPL15 |  |  | 98 | 0.215 | 0.1900 | Yes |
| 33 | RPS23 |  |  | 99 | 0.213 | 0.1953 | Yes |
| 34 | RPS7 |  |  | 107 | 0.210 | 0.1996 | Yes |
| 35 | EIF1B |  |  | 112 | 0.207 | 0.2043 | Yes |
| 36 | TP53 |  |  | 116 | 0.205 | 0.2090 | Yes |
| 37 | RPL12 |  |  | 121 | 0.203 | 0.2135 | Yes |
| 38 | PNRC1 |  |  | 123 | 0.202 | 0.2184 | Yes |
| 39 | PAPOLA |  |  | 131 | 0.199 | 0.2225 | Yes |
| 40 | RPS27A |  |  | 136 | 0.198 | 0.2269 | Yes |
| 41 | RPS24 |  |  | 137 | 0.197 | 0.2318 | Yes |
| 42 | H1FX |  |  | 145 | 0.194 | 0.2358 | Yes |
| 43 | SOX9 |  |  | 148 | 0.193 | 0.2403 | Yes |
| 44 | RPS2 |  |  | 156 | 0.189 | 0.2442 | Yes |
| 45 | RPS12 |  |  | 164 | 0.187 | 0.2480 | Yes |
| 46 | SRP14 |  |  | 181 | 0.182 | 0.2506 | Yes |
| 47 | RPS18 |  |  | 185 | 0.181 | 0.2547 | Yes |
| 48 | POLR2J |  |  | 190 | 0.180 | 0.2586 | Yes |
| 49 | RPL14 |  |  | 192 | 0.178 | 0.2629 | Yes |
| 50 | RPS15 |  |  | 193 | 0.178 | 0.2673 | Yes |
| 51 | DDIT3 |  |  | 205 | 0.175 | 0.2703 | Yes |
| 52 | TRIAP1 |  |  | 214 | 0.170 | 0.2736 | Yes |
| 53 | RPL8 |  |  | 215 | 0.170 | 0.2778 | Yes |
| 54 | ODC1 |  |  | 222 | 0.169 | 0.2813 | Yes |
| 55 | RPL10A |  |  | 233 | 0.166 | 0.2842 | Yes |
| 56 | RPL24 |  |  | 242 | 0.164 | 0.2873 | Yes |
| 57 | RPL23A |  |  | 246 | 0.163 | 0.2909 | Yes |
| 58 | TMSB15A |  |  | 253 | 0.161 | 0.2942 | Yes |
| 59 | RPL13A |  |  | 255 | 0.161 | 0.2981 | Yes |
| 60 | RSL24D1 |  |  | 264 | 0.159 | 0.3011 | Yes |
| 61 | ZFP36L1 |  |  | 266 | 0.159 | 0.3049 | Yes |
| 62 | HINT1 |  |  | 274 | 0.156 | 0.3079 | Yes |
| 63 | RPL9 |  |  | 290 | 0.153 | 0.3099 | Yes |
| 64 | WDR43 |  |  | 301 | 0.151 | 0.3124 | Yes |
| 65 | GNG5 |  |  | 326 | 0.147 | 0.3132 | Yes |
| 66 | GPX4 |  |  | 365 | 0.141 | 0.3122 | Yes |
| 67 | PPM1D |  |  | 386 | 0.139 | 0.3132 | Yes |
| 68 | CMBL |  |  | 402 | 0.136 | 0.3148 | Yes |
| 69 | SNRPE |  |  | 417 | 0.133 | 0.3164 | Yes |
| 70 | BTBD1 |  |  | 418 | 0.133 | 0.3197 | Yes |
| 71 | TCF7L1 |  |  | 427 | 0.131 | 0.3220 | Yes |
| 72 | YBX3 |  |  | 432 | 0.131 | 0.3247 | Yes |
| 73 | CCDC25 |  |  | 456 | 0.127 | 0.3251 | Yes |
| 74 | RPL23 |  |  | 457 | 0.127 | 0.3283 | Yes |
| 75 | ATF3 |  |  | 494 | 0.124 | 0.3270 | Yes |
| 76 | CAPZA1 |  |  | 502 | 0.123 | 0.3293 | Yes |
| 77 | RPS26 |  |  | 512 | 0.122 | 0.3312 | Yes |
| 78 | H3F3B |  |  | 514 | 0.122 | 0.3341 | Yes |
| 79 | ADNP |  |  | 517 | 0.121 | 0.3369 | Yes |
| 80 | RSRC2 |  |  | 535 | 0.120 | 0.3378 | Yes |
| 81 | RPL21 |  |  | 554 | 0.118 | 0.3385 | Yes |
| 82 | HDAC9 |  |  | 574 | 0.116 | 0.3392 | Yes |
| 83 | PUF60 |  |  | 600 | 0.114 | 0.3390 | Yes |
| 84 | PHLDA1 |  |  | 612 | 0.113 | 0.3405 | Yes |
| 85 | EAF1 |  |  | 628 | 0.112 | 0.3414 | Yes |
| 86 | RPS6 |  |  | 647 | 0.110 | 0.3420 | Yes |
| 87 | HMGN2 |  |  | 679 | 0.108 | 0.3410 | Yes |
| 88 | EEF1D |  |  | 682 | 0.107 | 0.3434 | Yes |
| 89 | IRF2BP2 |  |  | 709 | 0.105 | 0.3429 | Yes |
| 90 | LSM4 |  |  | 728 | 0.103 | 0.3433 | Yes |
| 91 | NDUFA6 |  |  | 730 | 0.103 | 0.3457 | Yes |
| 92 | RPL35 |  |  | 756 | 0.102 | 0.3452 | Yes |
| 93 | HIST1H1C |  |  | 768 | 0.101 | 0.3464 | Yes |
| 94 | ATF4 |  |  | 777 | 0.100 | 0.3479 | Yes |
| 95 | WBP4 |  |  | 789 | 0.099 | 0.3491 | Yes |
| 96 | ZNF217 |  |  | 798 | 0.098 | 0.3505 | Yes |
| 97 | HSPA1B |  |  | 814 | 0.097 | 0.3512 | Yes |
| 98 | ASNS |  |  | 850 | 0.094 | 0.3493 | No |
| 99 | SOX2 |  |  | 863 | 0.093 | 0.3502 | No |
| 100 | EZH2 |  |  | 940 | 0.089 | 0.3433 | No |
| 101 | PPP1R15A |  |  | 968 | 0.087 | 0.3422 | No |
| 102 | COPS8 |  |  | 975 | 0.087 | 0.3437 | No |
| 103 | HSPA4L |  |  | 1005 | 0.085 | 0.3423 | No |
| 104 | RPL7 |  |  | 1076 | 0.082 | 0.3360 | No |
| 105 | CTNNAL1 |  |  | 1078 | 0.082 | 0.3379 | No |
| 106 | EIF1AD |  |  | 1079 | 0.081 | 0.3399 | No |
| 107 | KIF5B |  |  | 1112 | 0.080 | 0.3381 | No |
| 108 | TBL1X |  |  | 1121 | 0.080 | 0.3391 | No |
| 109 | GLO1 |  |  | 1161 | 0.077 | 0.3363 | No |
| 110 | USP39 |  |  | 1198 | 0.075 | 0.3339 | No |
| 111 | BNIP3 |  |  | 1202 | 0.075 | 0.3354 | No |
| 112 | CDC42EP4 |  |  | 1204 | 0.075 | 0.3371 | No |
| 113 | ERRFI1 |  |  | 1210 | 0.075 | 0.3384 | No |
| 114 | CREB5 |  |  | 1231 | 0.074 | 0.3378 | No |
| 115 | PDCD4 |  |  | 1248 | 0.074 | 0.3378 | No |
| 116 | PIK3R3 |  |  | 1321 | 0.070 | 0.3309 | No |
| 117 | FXR1 |  |  | 1353 | 0.069 | 0.3289 | No |
| 118 | CKB |  |  | 1359 | 0.069 | 0.3300 | No |
| 119 | PSIP1 |  |  | 1372 | 0.068 | 0.3303 | No |
| 120 | SVIP |  |  | 1373 | 0.068 | 0.3320 | No |
| 121 | GSK3B |  |  | 1382 | 0.068 | 0.3327 | No |
| 122 | PPP4R2 |  |  | 1519 | 0.063 | 0.3180 | No |
| 123 | DEK |  |  | 1535 | 0.063 | 0.3177 | No |
| 124 | ACAA2 |  |  | 1540 | 0.062 | 0.3188 | No |
| 125 | TFAP2C |  |  | 1552 | 0.062 | 0.3190 | No |
| 126 | WTAP |  |  | 1561 | 0.062 | 0.3196 | No |
| 127 | PPP1CC |  |  | 1577 | 0.061 | 0.3193 | No |
| 128 | ARRDC3 |  |  | 1579 | 0.061 | 0.3207 | No |
| 129 | TIMELESS |  |  | 1584 | 0.061 | 0.3218 | No |
| 130 | TCF3 |  |  | 1619 | 0.060 | 0.3192 | No |
| 131 | DUSP16 |  |  | 1643 | 0.059 | 0.3179 | No |
| 132 | TAF9 |  |  | 1644 | 0.059 | 0.3193 | No |
| 133 | TJP1 |  |  | 1659 | 0.058 | 0.3191 | No |
| 134 | TOPORS |  |  | 1691 | 0.057 | 0.3168 | No |
| 135 | USP1 |  |  | 1712 | 0.056 | 0.3158 | No |
| 136 | AHSA1 |  |  | 1762 | 0.055 | 0.3113 | No |
| 137 | HSPE1 |  |  | 1803 | 0.054 | 0.3078 | No |
| 138 | SLC25A3 |  |  | 1835 | 0.052 | 0.3054 | No |
| 139 | PEG3 |  |  | 1846 | 0.052 | 0.3055 | No |
| 140 | RBM8A |  |  | 1852 | 0.052 | 0.3062 | No |
| 141 | ZCCHC3 |  |  | 1856 | 0.052 | 0.3071 | No |
| 142 | RHEB |  |  | 1873 | 0.051 | 0.3065 | No |
| 143 | SNRPG |  |  | 1951 | 0.049 | 0.2985 | No |
| 144 | CCT4 |  |  | 1969 | 0.049 | 0.2977 | No |
| 145 | KDM3A |  |  | 1991 | 0.048 | 0.2964 | No |
| 146 | BTG3 |  |  | 2012 | 0.047 | 0.2952 | No |
| 147 | IMMP1L |  |  | 2028 | 0.047 | 0.2945 | No |
| 148 | MIER1 |  |  | 2038 | 0.047 | 0.2946 | No |
| 149 | TCF7L2 |  |  | 2133 | 0.044 | 0.2845 | No |
| 150 | C1orf61 |  |  | 2155 | 0.043 | 0.2830 | No |
| 151 | SLIRP |  |  | 2169 | 0.043 | 0.2825 | No |
| 152 | NAP1L1 |  |  | 2171 | 0.043 | 0.2835 | No |
| 153 | RPL22L1 |  |  | 2183 | 0.042 | 0.2832 | No |
| 154 | RFX4 |  |  | 2238 | 0.040 | 0.2777 | No |
| 155 | TLK1 |  |  | 2244 | 0.040 | 0.2781 | No |
| 156 | TTK |  |  | 2262 | 0.040 | 0.2771 | No |
| 157 | OSER1 |  |  | 2271 | 0.039 | 0.2771 | No |
| 158 | PIN4 |  |  | 2295 | 0.039 | 0.2753 | No |
| 159 | PTTG1 |  |  | 2304 | 0.038 | 0.2753 | No |
| 160 | GSTP1 |  |  | 2453 | 0.035 | 0.2585 | No |
| 161 | MND1 |  |  | 2472 | 0.034 | 0.2572 | No |
| 162 | POLR2L |  |  | 2539 | 0.032 | 0.2501 | No |
| 163 | ZNF516 |  |  | 2551 | 0.032 | 0.2495 | No |
| 164 | ANP32E |  |  | 2570 | 0.032 | 0.2482 | No |
| 165 | MED30 |  |  | 2592 | 0.031 | 0.2464 | No |
| 166 | NOTCH3 |  |  | 2595 | 0.031 | 0.2470 | No |
| 167 | MSI1 |  |  | 2602 | 0.031 | 0.2470 | No |
| 168 | TMEM258 |  |  | 2608 | 0.031 | 0.2472 | No |
| 169 | HMGN3 |  |  | 2630 | 0.030 | 0.2454 | No |
| 170 | CASP3 |  |  | 2634 | 0.030 | 0.2458 | No |
| 171 | DPM1 |  |  | 2665 | 0.030 | 0.2429 | No |
| 172 | PTAR1 |  |  | 2800 | 0.027 | 0.2276 | No |
| 173 | SFRP1 |  |  | 2812 | 0.026 | 0.2269 | No |
| 174 | VIM |  |  | 2831 | 0.026 | 0.2254 | No |
| 175 | PSMD14 |  |  | 2855 | 0.025 | 0.2233 | No |
| 176 | UBB |  |  | 2890 | 0.024 | 0.2198 | No |
| 177 | MAP1B |  |  | 2893 | 0.024 | 0.2202 | No |
| 178 | TOMM5 |  |  | 2919 | 0.024 | 0.2178 | No |
| 179 | MYADM |  |  | 2933 | 0.023 | 0.2168 | No |
| 180 | EIF3J |  |  | 2953 | 0.023 | 0.2151 | No |
| 181 | CKS1B |  |  | 2985 | 0.022 | 0.2119 | No |
| 182 | SRSF3 |  |  | 3000 | 0.022 | 0.2108 | No |
| 183 | FOXN2 |  |  | 3044 | 0.021 | 0.2062 | No |
| 184 | NUP50 |  |  | 3103 | 0.020 | 0.1998 | No |
| 185 | NDUFB1 |  |  | 3119 | 0.020 | 0.1985 | No |
| 186 | REST |  |  | 3154 | 0.019 | 0.1948 | No |
| 187 | PBDC1 |  |  | 3178 | 0.018 | 0.1926 | No |
| 188 | PPA1 |  |  | 3215 | 0.017 | 0.1887 | No |
| 189 | HAUS8 |  |  | 3252 | 0.016 | 0.1848 | No |
| 190 | ZDHHC2 |  |  | 3261 | 0.016 | 0.1842 | No |
| 191 | YAP1 |  |  | 3295 | 0.016 | 0.1807 | No |
| 192 | SAR1A |  |  | 3350 | 0.014 | 0.1746 | No |
| 193 | HMGB3 |  |  | 3377 | 0.014 | 0.1718 | No |
| 194 | RBBP8 |  |  | 3390 | 0.013 | 0.1707 | No |
| 195 | TRA2A |  |  | 3410 | 0.013 | 0.1687 | No |
| 196 | CNN3 |  |  | 3415 | 0.013 | 0.1686 | No |
| 197 | RACGAP1 |  |  | 3432 | 0.012 | 0.1670 | No |
| 198 | TYW3 |  |  | 3457 | 0.012 | 0.1644 | No |
| 199 | SALL1 |  |  | 3467 | 0.012 | 0.1636 | No |
| 200 | HEY1 |  |  | 3541 | 0.011 | 0.1551 | No |
| 201 | CNIH1 |  |  | 3554 | 0.010 | 0.1540 | No |
| 202 | TNFRSF19 |  |  | 3571 | 0.010 | 0.1523 | No |
| 203 | HSPB1 |  |  | 3587 | 0.010 | 0.1507 | No |
| 204 | DUT |  |  | 3596 | 0.009 | 0.1500 | No |
| 205 | HIST1H1E |  |  | 3635 | 0.008 | 0.1457 | No |
| 206 | THUMPD1 |  |  | 3657 | 0.008 | 0.1434 | No |
| 207 | TUBE1 |  |  | 3670 | 0.007 | 0.1421 | No |
| 208 | TGIF1 |  |  | 3672 | 0.007 | 0.1422 | No |
| 209 | TPM1 |  |  | 3680 | 0.007 | 0.1415 | No |
| 210 | DHX40 |  |  | 3724 | 0.006 | 0.1365 | No |
| 211 | RNPS1 |  |  | 3725 | 0.006 | 0.1367 | No |
| 212 | TCP1 |  |  | 3757 | 0.006 | 0.1331 | No |
| 213 | TAF7 |  |  | 3759 | 0.006 | 0.1331 | No |
| 214 | NEK4 |  |  | 3770 | 0.005 | 0.1321 | No |
| 215 | NOP58 |  |  | 3860 | 0.003 | 0.1215 | No |
| 216 | FAM162A |  |  | 3867 | 0.003 | 0.1209 | No |
| 217 | CENPW |  |  | 3936 | 0.002 | 0.1128 | No |
| 218 | YWHAE |  |  | 3963 | 0.001 | 0.1097 | No |
| 219 | PPIA |  |  | 3964 | 0.001 | 0.1097 | No |
| 220 | FDFT1 |  |  | 3979 | 0.001 | 0.1081 | No |
| 221 | TSPAN6 |  |  | 3995 | 0.001 | 0.1063 | No |
| 222 | RC3H2 |  |  | 4000 | 0.001 | 0.1059 | No |
| 223 | RDH10 |  |  | 4024 | 0.000 | 0.1031 | No |
| 224 | GPN1 |  |  | 4027 | 0.000 | 0.1029 | No |
| 225 | RYBP |  |  | 4056 | -0.000 | 0.0995 | No |
| 226 | GPCPD1 |  |  | 4087 | -0.001 | 0.0960 | No |
| 227 | ID4 |  |  | 4102 | -0.001 | 0.0943 | No |
| 228 | IFT74 |  |  | 4114 | -0.002 | 0.0931 | No |
| 229 | KCTD6 |  |  | 4200 | -0.003 | 0.0830 | No |
| 230 | CBFB |  |  | 4209 | -0.003 | 0.0821 | No |
| 231 | ZNF134 |  |  | 4223 | -0.004 | 0.0806 | No |
| 232 | MDK |  |  | 4311 | -0.005 | 0.0704 | No |
| 233 | CEP135 |  |  | 4341 | -0.006 | 0.0671 | No |
| 234 | SNRPB |  |  | 4387 | -0.007 | 0.0618 | No |
| 235 | UQCRFS1 |  |  | 4466 | -0.008 | 0.0527 | No |
| 236 | SNRPD1 |  |  | 4477 | -0.008 | 0.0517 | No |
| 237 | HMGA1 |  |  | 4523 | -0.009 | 0.0466 | No |
| 238 | EFNB1 |  |  | 4526 | -0.009 | 0.0466 | No |
| 239 | LDHB |  |  | 4572 | -0.010 | 0.0414 | No |
| 240 | INTS8 |  |  | 4602 | -0.011 | 0.0382 | No |
| 241 | SEMA5A |  |  | 4642 | -0.012 | 0.0339 | No |
| 242 | RAP2C |  |  | 4662 | -0.012 | 0.0319 | No |
| 243 | CCNB2 |  |  | 4736 | -0.013 | 0.0235 | No |
| 244 | UHRF1 |  |  | 4792 | -0.014 | 0.0173 | No |
| 245 | KIAA1217 |  |  | 4799 | -0.014 | 0.0169 | No |
| 246 | DDX39A |  |  | 4809 | -0.014 | 0.0162 | No |
| 247 | KIF14 |  |  | 4913 | -0.017 | 0.0043 | No |
| 248 | RTKN2 |  |  | 4914 | -0.017 | 0.0047 | No |
| 249 | ZFHX4 |  |  | 4941 | -0.017 | 0.0020 | No |
| 250 | CSNK2B |  |  | 5064 | -0.020 | -0.0121 | No |
| 251 | ZFP36L2 |  |  | 5069 | -0.020 | -0.0121 | No |
| 252 | ADAMTS9 |  |  | 5090 | -0.020 | -0.0140 | No |
| 253 | ZNF844 |  |  | 5194 | -0.022 | -0.0257 | No |
| 254 | DPY30 |  |  | 5212 | -0.022 | -0.0272 | No |
| 255 | CHMP1B |  |  | 5220 | -0.022 | -0.0275 | No |
| 256 | LEF1 |  |  | 5275 | -0.024 | -0.0333 | No |
| 257 | MTHFD2L |  |  | 5314 | -0.025 | -0.0373 | No |
| 258 | FKBP10 |  |  | 5319 | -0.025 | -0.0371 | No |
| 259 | GPBP1L1 |  |  | 5352 | -0.025 | -0.0403 | No |
| 260 | HIST1H1B |  |  | 5374 | -0.026 | -0.0422 | No |
| 261 | NPM1 |  |  | 5412 | -0.027 | -0.0460 | No |
| 262 | TIPARP |  |  | 5417 | -0.027 | -0.0458 | No |
| 263 | SNRPA1 |  |  | 5428 | -0.027 | -0.0463 | No |
| 264 | PBX3 |  |  | 5501 | -0.028 | -0.0542 | No |
| 265 | HMGA2 |  |  | 5509 | -0.029 | -0.0544 | No |
| 266 | ECT2 |  |  | 5560 | -0.030 | -0.0596 | No |
| 267 | SSB |  |  | 5596 | -0.030 | -0.0630 | No |
| 268 | MRPL47 |  |  | 5614 | -0.031 | -0.0643 | No |
| 269 | VEGFA |  |  | 5660 | -0.032 | -0.0689 | No |
| 270 | ZFAND2A |  |  | 5753 | -0.034 | -0.0791 | No |
| 271 | SP3 |  |  | 5802 | -0.034 | -0.0839 | No |
| 272 | KNSTRN |  |  | 5875 | -0.036 | -0.0917 | No |
| 273 | BET1 |  |  | 5949 | -0.037 | -0.0995 | No |
| 274 | CREB3L2 |  |  | 5989 | -0.038 | -0.1032 | No |
| 275 | NUP155 |  |  | 6072 | -0.040 | -0.1120 | No |
| 276 | H2AFZ |  |  | 6079 | -0.040 | -0.1118 | No |
| 277 | CYCS |  |  | 6090 | -0.040 | -0.1120 | No |
| 278 | AHI1 |  |  | 6098 | -0.040 | -0.1118 | No |
| 279 | SPC24 |  |  | 6135 | -0.041 | -0.1151 | No |
| 280 | SPAG5 |  |  | 6146 | -0.041 | -0.1153 | No |
| 281 | ANAPC16 |  |  | 6169 | -0.042 | -0.1168 | No |
| 282 | PSMB7 |  |  | 6178 | -0.042 | -0.1168 | No |
| 283 | CENPN |  |  | 6194 | -0.042 | -0.1175 | No |
| 284 | CCDC59 |  |  | 6252 | -0.044 | -0.1232 | No |
| 285 | CHORDC1 |  |  | 6278 | -0.044 | -0.1251 | No |
| 286 | BARD1 |  |  | 6284 | -0.044 | -0.1246 | No |
| 287 | CDKN3 |  |  | 6291 | -0.044 | -0.1242 | No |
| 288 | PHLPP1 |  |  | 6292 | -0.044 | -0.1231 | No |
| 289 | CEP152 |  |  | 6298 | -0.044 | -0.1226 | No |
| 290 | RNF139 |  |  | 6328 | -0.045 | -0.1250 | No |
| 291 | KIF18A |  |  | 6544 | -0.051 | -0.1494 | No |
| 292 | MDM1 |  |  | 6590 | -0.053 | -0.1535 | No |
| 293 | PTGES3 |  |  | 6602 | -0.053 | -0.1535 | No |
| 294 | NFYB |  |  | 6609 | -0.053 | -0.1529 | No |
| 295 | HSPH1 |  |  | 6617 | -0.053 | -0.1524 | No |
| 296 | HSP90AB1 |  |  | 6619 | -0.053 | -0.1512 | No |
| 297 | SCAF11 |  |  | 6653 | -0.054 | -0.1538 | No |
| 298 | ETF1 |  |  | 6699 | -0.056 | -0.1578 | No |
| 299 | SCAND1 |  |  | 6709 | -0.056 | -0.1575 | No |
| 300 | BUB1B |  |  | 6717 | -0.056 | -0.1570 | No |
| 301 | PGAM1 |  |  | 6725 | -0.057 | -0.1564 | No |
| 302 | MKI67 |  |  | 6750 | -0.057 | -0.1578 | No |
| 303 | PPIL4 |  |  | 6810 | -0.059 | -0.1634 | No |
| 304 | MYL6 |  |  | 6811 | -0.059 | -0.1620 | No |
| 305 | RBM39 |  |  | 6825 | -0.059 | -0.1621 | No |
| 306 | GPC4 |  |  | 6884 | -0.061 | -0.1675 | No |
| 307 | FANCD2 |  |  | 6903 | -0.062 | -0.1681 | No |
| 308 | PRC1 |  |  | 6918 | -0.062 | -0.1682 | No |
| 309 | CCDC18 |  |  | 6989 | -0.064 | -0.1750 | No |
| 310 | GLI3 |  |  | 7020 | -0.066 | -0.1770 | No |
| 311 | BIRC5 |  |  | 7024 | -0.066 | -0.1757 | No |
| 312 | NFKBIA |  |  | 7027 | -0.066 | -0.1743 | No |
| 313 | SIVA1 |  |  | 7054 | -0.066 | -0.1758 | No |
| 314 | CKAP2L |  |  | 7065 | -0.067 | -0.1753 | No |
| 315 | TWISTNB |  |  | 7084 | -0.067 | -0.1758 | No |
| 316 | SRPRB |  |  | 7090 | -0.067 | -0.1747 | No |
| 317 | HIST1H1D |  |  | 7093 | -0.068 | -0.1733 | No |
| 318 | KIF22 |  |  | 7098 | -0.068 | -0.1721 | No |
| 319 | LIFR |  |  | 7101 | -0.068 | -0.1707 | No |
| 320 | CCNL1 |  |  | 7107 | -0.068 | -0.1696 | No |
| 321 | G3BP1 |  |  | 7109 | -0.068 | -0.1680 | No |
| 322 | SLC3A2 |  |  | 7118 | -0.068 | -0.1673 | No |
| 323 | DNAJC1 |  |  | 7129 | -0.069 | -0.1668 | No |
| 324 | TMEM170A |  |  | 7160 | -0.070 | -0.1686 | No |
| 325 | HMGB1 |  |  | 7186 | -0.071 | -0.1699 | No |
| 326 | AGO3 |  |  | 7207 | -0.071 | -0.1705 | No |
| 327 | RAN |  |  | 7221 | -0.072 | -0.1703 | No |
| 328 | RAD51AP1 |  |  | 7236 | -0.072 | -0.1702 | No |
| 329 | MIS18BP1 |  |  | 7253 | -0.073 | -0.1703 | No |
| 330 | OFD1 |  |  | 7259 | -0.073 | -0.1691 | No |
| 331 | PTBP1 |  |  | 7293 | -0.074 | -0.1712 | No |
| 332 | HES1 |  |  | 7301 | -0.074 | -0.1702 | No |
| 333 | DARS |  |  | 7367 | -0.077 | -0.1760 | No |
| 334 | ZWINT |  |  | 7370 | -0.077 | -0.1744 | No |
| 335 | IER5 |  |  | 7383 | -0.077 | -0.1739 | No |
| 336 | WDR36 |  |  | 7428 | -0.079 | -0.1772 | No |
| 337 | TOP2A |  |  | 7430 | -0.079 | -0.1754 | No |
| 338 | HSPD1 |  |  | 7433 | -0.079 | -0.1737 | No |
| 339 | SPC25 |  |  | 7522 | -0.082 | -0.1821 | No |
| 340 | ENO1 |  |  | 7559 | -0.084 | -0.1844 | No |
| 341 | PRDX6 |  |  | 7589 | -0.085 | -0.1857 | No |
| 342 | SLC5A3 |  |  | 7592 | -0.086 | -0.1838 | No |
| 343 | MCM3 |  |  | 7597 | -0.086 | -0.1822 | No |
| 344 | SYNE2 |  |  | 7598 | -0.086 | -0.1801 | No |
| 345 | KIF11 |  |  | 7619 | -0.087 | -0.1803 | No |
| 346 | ZEB1 |  |  | 7621 | -0.087 | -0.1783 | No |
| 347 | DCLRE1C |  |  | 7627 | -0.087 | -0.1767 | No |
| 348 | CDCA3 |  |  | 7631 | -0.087 | -0.1749 | No |
| 349 | BOC |  |  | 7635 | -0.087 | -0.1731 | No |
| 350 | DBF4 |  |  | 7639 | -0.088 | -0.1713 | No |
| 351 | HIST1H4C |  |  | 7641 | -0.088 | -0.1693 | No |
| 352 | GMNN |  |  | 7651 | -0.088 | -0.1682 | No |
| 353 | PSMA1 |  |  | 7678 | -0.090 | -0.1690 | No |
| 354 | PKM |  |  | 7732 | -0.093 | -0.1731 | No |
| 355 | NDC80 |  |  | 7734 | -0.093 | -0.1709 | No |
| 356 | TPI1 |  |  | 7757 | -0.093 | -0.1712 | No |
| 357 | RPF2 |  |  | 7773 | -0.094 | -0.1707 | No |
| 358 | DYRK1A |  |  | 7781 | -0.094 | -0.1692 | No |
| 359 | ARHGEF10 |  |  | 7788 | -0.095 | -0.1676 | No |
| 360 | ATP6V0E1 |  |  | 7802 | -0.096 | -0.1668 | No |
| 361 | CHEK1 |  |  | 7852 | -0.099 | -0.1702 | No |
| 362 | PSMA3 |  |  | 7871 | -0.100 | -0.1699 | No |
| 363 | TAF8 |  |  | 7874 | -0.100 | -0.1676 | No |
| 364 | MCM4 |  |  | 7900 | -0.101 | -0.1681 | No |
| 365 | PBK |  |  | 7905 | -0.101 | -0.1661 | No |
| 366 | CCNB1 |  |  | 7958 | -0.104 | -0.1697 | No |
| 367 | ITGB3BP |  |  | 7959 | -0.104 | -0.1671 | No |
| 368 | NCAPG2 |  |  | 7976 | -0.105 | -0.1664 | No |
| 369 | RRM2 |  |  | 8001 | -0.107 | -0.1666 | No |
| 370 | MAGED2 |  |  | 8009 | -0.108 | -0.1648 | No |
| 371 | CACYBP |  |  | 8012 | -0.108 | -0.1624 | No |
| 372 | NOL7 |  |  | 8017 | -0.108 | -0.1602 | No |
| 373 | TMEM97 |  |  | 8049 | -0.110 | -0.1612 | No |
| 374 | WEE1 |  |  | 8054 | -0.111 | -0.1589 | No |
| 375 | PCNA |  |  | 8061 | -0.111 | -0.1569 | No |
| 376 | RBL1 |  |  | 8069 | -0.111 | -0.1550 | No |
| 377 | CKS2 |  |  | 8077 | -0.112 | -0.1530 | No |
| 378 | ATP1B1 |  |  | 8091 | -0.113 | -0.1518 | No |
| 379 | JAM3 |  |  | 8130 | -0.116 | -0.1535 | No |
| 380 | CNTLN |  |  | 8150 | -0.117 | -0.1528 | No |
| 381 | HSP90AA1 |  |  | 8153 | -0.117 | -0.1502 | No |
| 382 | PPP1R15B |  |  | 8155 | -0.117 | -0.1474 | No |
| 383 | FGFR3 |  |  | 8163 | -0.118 | -0.1453 | No |
| 384 | PON2 |  |  | 8177 | -0.120 | -0.1439 | No |
| 385 | ANLN |  |  | 8181 | -0.120 | -0.1413 | No |
| 386 | ITGB8 |  |  | 8195 | -0.121 | -0.1398 | No |
| 387 | XPO1 |  |  | 8216 | -0.123 | -0.1392 | No |
| 388 | CCNA2 |  |  | 8227 | -0.124 | -0.1373 | No |
| 389 | KIF15 |  |  | 8228 | -0.124 | -0.1342 | No |
| 390 | LDHA |  |  | 8261 | -0.127 | -0.1349 | No |
| 391 | TYMS |  |  | 8287 | -0.131 | -0.1347 | No |
| 392 | KIF4A |  |  | 8300 | -0.132 | -0.1328 | No |
| 393 | MRPL50 |  |  | 8327 | -0.135 | -0.1326 | No |
| 394 | KIF2C |  |  | 8328 | -0.135 | -0.1292 | No |
| 395 | CKAP2 |  |  | 8330 | -0.136 | -0.1260 | No |
| 396 | HIST1H1A |  |  | 8345 | -0.137 | -0.1243 | No |
| 397 | CDK1 |  |  | 8351 | -0.139 | -0.1214 | No |
| 398 | ATAD2 |  |  | 8359 | -0.141 | -0.1188 | No |
| 399 | TPX2 |  |  | 8370 | -0.143 | -0.1164 | No |
| 400 | HMGB2 |  |  | 8388 | -0.145 | -0.1149 | No |
| 401 | NCAPG |  |  | 8390 | -0.145 | -0.1114 | No |
| 402 | CENPU |  |  | 8403 | -0.147 | -0.1092 | No |
| 403 | PSMB3 |  |  | 8416 | -0.148 | -0.1070 | No |
| 404 | MAD2L1 |  |  | 8427 | -0.150 | -0.1045 | No |
| 405 | DLGAP5 |  |  | 8447 | -0.152 | -0.1030 | No |
| 406 | KIF23 |  |  | 8465 | -0.154 | -0.1012 | No |
| 407 | IGF2BP2 |  |  | 8487 | -0.158 | -0.0998 | No |
| 408 | UBE2T |  |  | 8489 | -0.159 | -0.0960 | No |
| 409 | ARHGAP11A |  |  | 8513 | -0.164 | -0.0947 | No |
| 410 | NUSAP1 |  |  | 8523 | -0.167 | -0.0916 | No |
| 411 | ABHD3 |  |  | 8575 | -0.179 | -0.0933 | No |
| 412 | KIF20B |  |  | 8576 | -0.180 | -0.0888 | No |
| 413 | PRR11 |  |  | 8580 | -0.182 | -0.0847 | No |
| 414 | SREK1 |  |  | 8636 | -0.206 | -0.0862 | No |
| 415 | ASPM |  |  | 8657 | -0.215 | -0.0832 | No |
| 416 | SMC2 |  |  | 8658 | -0.216 | -0.0779 | No |
| 417 | BRCA1 |  |  | 8659 | -0.217 | -0.0725 | No |
| 418 | CENPK |  |  | 8666 | -0.220 | -0.0678 | No |
| 419 | SPCS1 |  |  | 8668 | -0.222 | -0.0624 | No |
| 420 | SLC25A37 |  |  | 8671 | -0.224 | -0.0571 | No |
| 421 | FAT1 |  |  | 8679 | -0.228 | -0.0523 | No |
| 422 | TTYH1 |  |  | 8683 | -0.230 | -0.0470 | No |
| 423 | CENPF |  |  | 8703 | -0.240 | -0.0433 | No |
| 424 | HELLS |  |  | 8720 | -0.260 | -0.0388 | No |
| 425 | MCAM |  |  | 8727 | -0.266 | -0.0329 | No |
| 426 | CENPE |  |  | 8734 | -0.270 | -0.0270 | No |
| 427 | UBE2C |  |  | 8748 | -0.286 | -0.0214 | No |
| 428 | MDM4 |  |  | 8753 | -0.290 | -0.0147 | No |
| 429 | SMC4 |  |  | 8764 | -0.307 | -0.0083 | No |
| 430 | HSP90B1 |  |  | 8794 | -0.487 | 0.0002 | No |
Table: GSEA details [plain text format]

  

Fig 2: RICHARDS\_NOWAKOWSKI\_SCIENCE\_2017\_RG\_EARLY\_UPREG: Random ES distribution      
 Gene set null distribution of ES for **RICHARDS\_NOWAKOWSKI\_SCIENCE\_2017\_RG\_EARLY\_UPREG**
